# Mortality from neglected tropical diseases in Brazil, 2000–2011

**DOI:** 10.2471/BLT.15.152363

**Published:** 2015-11-24

**Authors:** Francisco Rogerlândio Martins-Melo, Alberto Novaes Ramos, Carlos Henrique Alencar, Jorg Heukelbach

**Affiliations:** a Department of Community Health, School of Medicine, Federal University of Ceará, Rua Professor Costa Mendes, 1608, 5. andar, Rodolfo Teófilo, 60430-140 Fortaleza, Ceará, Brazil.

## Abstract

**Objective:**

To describe mortality from neglected tropical diseases (NTDs) in Brazil, 2000–2011.

**Methods:**

We extracted information on cause of death, age, sex, ethnicity and place of residence from the nationwide mortality information system at the Brazilian Ministry of Health. We selected deaths in which the underlying cause of death was a neglected tropical disease (NTD), as defined by the World Health Organization (WHO) and based on its *International statistical classification of diseases and related health problems*, 10th revision (ICD-10) codes. For specific NTDs, we estimated crude and age-adjusted mortality rates and 95% confidence intervals (CI). We calculated crude and age-adjusted mortality rates and mortality rate ratios by age, sex, ethnicity and geographic area.

**Findings:**

Over the 12-year study period, 12 491 280 deaths were recorded; 76 847 deaths (0.62%) were caused by NTDs. Chagas disease was the most common cause of death (58 928 deaths; 76.7%), followed by schistosomiasis (6319 deaths; 8.2%) and leishmaniasis (3466 deaths; 4.5%). The average annual age-adjusted mortality from all NTDs combined was 4.30 deaths per 100 000 population (95% CI: 4.21–4.40). Rates were higher in males: 4.98 deaths per 100 000; people older than 69 years: 33.12 deaths per 100 000; Afro-Brazilians: 5.25 deaths per 100 000; and residents in the central-west region: 14.71 deaths per 100 000.

**Conclusion:**

NTDs are important causes of death and are a significant public health problem in Brazil. There is a need for intensive integrated control measures in areas of high morbidity and mortality.

## Résumé

**Objectif:**

Décrire la mortalité due aux maladies tropicales négligées au Brésil sur la période 2000–2011.

**Méthodes:**

Nous avons prélevé des informations sur la cause des décès, l'âge, le sexe, l'origine ethnique et le lieu de résidence dans le système d'information national sur la mortalité du ministère de la Santé brésilien. Nous avons sélectionné les décès pour lesquels la cause sous-jacente était une maladie tropicale négligée, au sens de la définition de l'Organisation mondiale de la Santé (OMS) et selon les codes de sa Classification statistique internationale des maladies et des problèmes de santé connexes, 10e révision (CIM-10). Nous avons estimé le taux de mortalité brut et ajusté en fonction de l'âge ainsi que l'intervalle de confiance (IC) de 95% relatifs à des maladies tropicales négligées spécifiques. Nous avons calculé le taux de mortalité brut et ajusté en fonction de l'âge ainsi que les ratios de taux de mortalité par âge, sexe, origine ethnique et situation géographique.

**Résultats:**

Sur la période de 12 années étudiée, 12 491 280 décès ont été enregistrés; 76 847 de ces décès (0,62%) étaient dus à des maladies tropicales négligées. La maladie de Chagas était la cause de décès la plus courante (58 928 décès; 76,7%), suivie de la schistosomiase (6319 décès; 8,2%) et de la leishmaniose (3466 décès; 4,5%). La mortalité annuelle moyenne ajustée en fonction de l'âge due à l'ensemble des maladies tropicales négligées était de 4,30 décès pour 100 000 personnes (IC 95%: 4,21-4,40). Le taux était plus élevé chez les hommes: 4,98 décès pour 100 000 personnes; les personnes de plus de 69 ans: 33,12 décès pour 100 000 personnes; les Afro-Brésiliens: 5,25 décès pour 100 000 personnes; et les habitants de la région Centre-Ouest: 14,71 décès pour 100 000 personnes.

**Conclusion:**

Les maladies tropicales négligées représentent des causes de décès importantes et un grave problème de santé publique au Brésil. Des mesures de lutte intégrées et intensives sont nécessaires dans les régions qui présentent une morbidité et une mortalité élevées.

## Resumen

**Objetivo:**

Describir la mortalidad de las enfermedades tropicales desatendidas en Brasil, 2000–2011.

**Métodos:**

Se extrajo información referente a la causa del fallecimiento, edad, sexo, etnia y lugar de residencia del sistema de información de la mortalidad nacional del Ministerio de Salud de Brasil. Se seleccionaron fallecimientos en los que la causa subyacente de la muerte fue una enfermedad tropical desatendida, según las define la Organización Mundial de la Salud (OMS) y en base a los códigos de la Décima Revisión de la Clasificación Estadística Internacional de Enfermedades y Problemas Relacionados con la Salud (CIE-10). En el caso de enfermedades tropicales desatendidas concretas, se estimaron las tasas de mortalidad brutas y ajustadas por edades y los intervalos de confianza (IC) del 95%. Se calcularon las tasas de mortalidad brutas y ajustadas por edades y las razones de tasas de mortalidad ajustadas por edad, sexo, etnia y zona geográfica.

**Resultados:**

Durante el periodo de estudio de 12 años, se registraron 12 491 280 fallecimientos; 76 847 fallecimientos (0,62%) fueron causados por enfermedades tropicales desatendidas. La causa de fallecimiento más común fue la enfermedad de Chagas (58 928 fallecimientos; 76,7%), seguida de la esquistosomiasis (6319 fallecimientos; 8,2%) y la leishmaniasis (3466 fallecimientos; 4,5%). La media de mortalidad anual ajustada por edades de todas las enfermedades tropicales desatendidas combinadas fue de 4,30 fallecimientos por cada 100 000 habitantes (IC del 95%: 4,21–4,40). Las tasas fueron más altas en los hombres: 4,98 fallecimientos por cada 100 000 habitantes; personas mayores de 69 años: 33,12 fallecimientos por cada 100 000 habitantes; afrobrasileños: 5,25 fallecimientos por cada 100 000 habitantes; y residentes en la región centro-oeste: 14,71 fallecimientos por cada 100 000 habitantes.

**Conclusión:**

Las enfermedades tropicales desatendidas son importantes causas de fallecimiento y son un problema de salud pública significativo en Brasil. Existe la necesidad de tomar medidas de control intensivas integradas en zonas de morbilidad y mortalidad altas.

## ملخص

**الغرض:**

وصف معدّل الوفيات الناتجة عن الأمراض المدارية التي لا تلقى الاهتمام اللازم (NTD) في البرازيل، في الفترة من عام 2000 إلى عام 2011.

**الطريقة:**

قمنا باستخلاص المعلومات المتعلقة بسبب الوفاة والعمر والنوع والعِرق ومحل الإقامة من النظام الوطني للمعلومات عن الوفيات التابع لوزارة الصحة البرازيلية. وقد اخترنا الوفيات التي وقعت نتيجة لأحد الأمراض المدارية التي لا تلقى الاهتمام اللازم (NTD) وفقًا للتعريف الصادر عن منظمة الصحة العالمية، وبناءً على الرموز الواردة في تصنيفها الدولي الإحصائي للأمراض والمشاكل الصحية ذات الصلة، في المراجعة العاشرة (ICD-10) لها. أما بالنسبة لحالات محددة من الأمراض المدارية التي لا تلقى الاهتمام اللازم (NTD)، قمنا بتقدير معدلات الوفيات الخام ومعدلات الوفيات المعدّلة بحسب الأعمار ونسب الأرجحية (CI) بمقدار 95%. وقمنا بحساب معدلات الوفيات الخام ومعدلات الوفيات المعدّلة بحسب الأعمار ونسب معدلات الوفيات بحسب العمر، والنوع، والعِرق، والموقع الجغرافي.

**النتائج:**

في خلال فترة الدراسة التي أجريت على مدار 12 عامًا، تم تسجيل 12 491 280 حالة وفاة؛ وقد نتجت 76 847 حالة وفاة (بنسبة 0.62%) عن الأمراض المدارية التي لا تلقى الاهتمام اللازم. وكان مرض شاغاس هو سبب الوفاة الأكثر شيوعًا (بمعدل 58 928 حالة وفاة؛ وبنسبة 76.7%)، يليه داء البلهارسيا (بمعدل 6319 حالة وفاة؛ ونسبة 8.2%)، وداء الليشمانيات (بمعدل 3466 حالة وفاة؛ ونسبة 4.5%). وكان متوسط معدل الوفيات المعدّلة بحسب الأعمار والناتجة عن جميع الأمراض المدارية التي لا تلقى الاهتمام اللازم مجتمعة هو 4.30 حالة وفاة من بين 100 000 شخص من السكان (بنسبة أرجحية مقدارها 95%: 4.21–4.40). كانت المعدلات أكبر بين الذكور: 4.98 حالة وفاة من بين 100 000 شخص؛ الأشخاص الأكبر من 69 عامًا: 33.12 حالة وفاة من بين 100 000 شخص؛ البرازيليون من أصول أفريقية: 5.25 حالة وفاة من بين 100 000 شخص؛ والمقيمون في المنطقة المركزية الغربية: 14.71 حالة وفاة من بين 100 000 شخص.

**الاستنتاج:**

تعد الأمراض المدارية التي لا تلقى الاهتمام اللازم من ضمن الأسباب المهمة لحدوث الوفيات وتمثل مشكلة صحية عامة كبيرة في البرازيل. وهناك حاجة لاتخاذ تدابير المكافحة المتكاملة المكثفة في المناطق التي تعاني من معدلات عالية من الإصابة بالأمراض والوفيات.

## 摘要

**目的:**

旨在描述 2000 年至 2011 年巴西被忽视热带疾病 (NTDs) 死亡率。

**方法:**

我们从巴西卫生部全国死亡率系统提取死亡、年龄、性别、种族和居住地信息。并根据世界卫生组织的相关界定和《国际疾病伤害及死因分类标准》第 10 版 (ICD-10) 中内容，选取死亡原因为被忽视热带疾病 (NTDs) 的死亡案例。对于特定的被忽视热带疾病 (NTDs)，我们估计其年龄标准化粗死亡率，和 95% 置信区间 (CI)。并计算了年龄标准化粗死亡率和按年龄、性别、种族和地理区域划分的死亡率。

**结果:**

通过 12 年的研究，共记录了 12 491 280 例死亡；其中 76 847 例是由被忽视热带疾病 (NTDs) 引起的。恰加斯病是最常见的死亡原因（58 928 例；76.7%）；其次是血吸虫病（6319 例；8.2%）和利什曼病（3466 例；4.5%）。所有涉及被忽视热带疾病 (NTDs) 的年龄标准化平均年死亡率是每 100000 例占 4.3 例 95% 置信区间 (CI):4.21–4.40). 男性死亡率更高：每 100000 例占 4.98 例；69 岁以上人群：每 100000 例占 33.12 例；非裔巴西人：每 100000 例占 5.25 例；中西部地区：每 100000 例占 14.71 例。

**结论:**

被忽视热带疾病 (NTDs) 是巴西重要的死亡原因，而且是非常严重的公众健康问题。需加强对高发病率和高死亡率地区的综合措施管控。

## Резюме

**Цель:**

Определить смертность от остающихся без внимания тропических болезней (ОВТБ) в Бразилии за период 2000–2011 гг.

**Методы:**

Из национальной информационной системы по смертности Министерства здравоохранения Бразилии была получена информация о причине смерти, возрасте, поле, национальности и месте проживания умершего. Были выбраны смерти, исходной причиной которых были остающиеся без внимания тропические болезни (ОВТБ) в соответствии с определением Всемирной организации здравоохранения (ВОЗ) и на основании ее *Международной статистической классификации болезней и проблем, связанных со здоровьем*, 10-е  переработанное и исправленное издание (МКБ-10). Для отдельных ОВТБ были подсчитаны общий и стандартизированный по возрасту уровни смертности и 95%-е доверительные интервалы (ДИ). Были подсчитаны общий и стандартизированный по возрасту уровни смертности и коэффициенты смертности по возрасту, полу, национальности и географическому району.

**Результаты:**

За 12-летний период исследования было зарегистрировано 12 491 280 смертей; причиной 76 847 смертей (0,62%) были ОВТБ. Болезнь Шагаса была наиболее распространенной причиной смерти (58 928 смертей; 76,7%), менее распространенными были шистосомоз (6319 смертей; 8,2%) и лейшманиоз (3466 смертей; 4,5%). Средняя годовая стандартизированная по возрасту смертность от всех ОВТБ в совокупности составила 4,3 смерти на 100 000 человек населения (95%-й ДИ: 4,21–4,40). Уровень смертности был выше среди мужчин: 4,98 смерти на 100 000; среди людей в возрасте старше 69 лет: 33,12 смерти на 100 000; среди афробразильцев: 5,25 смерти на 100 000; среди жителей Центрально-Западного региона: 14,71 смерти на 100 000.

## Introduction

Neglected tropical diseases (NTDs) can result in disabilities, disfigurement, impaired childhood growth and cognitive development, death and increasing poverty in affected communities.^1^ Worldwide, about 2 billion people are at risk of one or more NTDs and more than 1 billion people are affected by these diseases.^1^^–^^3^ Up to half a million deaths and up to 57 million disability-adjusted life years lost have been attributed annually to NTDs.^1^^,^^2^^,^^4^^,^^5^

Brazil accounts for a large proportion of NTDs occurring in Latin America, including leprosy (86%), dengue fever (40%), schistosomiasis (96%), Chagas disease (25%), cutaneous leishmaniasis (39%) and visceral leishmaniasis (93%).^6^^–^^8^ Most NTDs occur in populations with low-socioeconomic status, mainly in the north and north-east of the country.^6^

Knowledge of the magnitude of NTD-related deaths in endemic countries is essential for monitoring and evaluation of the impact of interventions and the effectiveness of specific control measures.^9^^–^^11^ However, there are only a few systematic and large-scale studies investigating NTD-related mortality.^9^^,^^10^^,^^12^^–^^16^ Here, we describe the epidemiological characteristics of deaths due to NTDs in Brazil over a period of 12 years.

## Methods

We obtained mortality data from the nationwide mortality information system of the Brazilian Ministry of Health, which is publicly accessible.^17^ Death certificates, which are completed by physicians, include the following variables: multiple causes of death, age, sex, education, ethnicity, marital status, date of death, place of residence and place of death. We downloaded and processed a total of 324 mortality data sets (one for each of the 27 states per year). We included all deaths in Brazil from 2000 to 2011, in which any NTD was recorded on death certificates as the underlying cause of death. We selected all NTDs as defined by the World Health Organization (WHO) based on its *International statistical classification of diseases and related health problems,* 10th revision (ICD-10) codes,^18^ whether or not the disease is known to be endemic in Brazil (Table 1).^1^^,^^4^ Population data were based on the national population censuses (2000 and 2010) with interpolation for other years (2001–2009 and 2011).^19^

**Table 1:** Neglected tropical diseases defined by the World Health Organization, recorded in the mortality information system, Brazil, 2000–2011

Disease	ICD-10 code	Endemic in Brazil^a^
**Protozoa**		
Chagas disease (American trypanosomiasis)	B57	Yes
Leishmaniasis	B55	Yes
Human African trypanosomiasis (sleeping sickness)	B56	No
**Helminths**		
Schistosomiasis	B65	Yes
Soil-transmitted helminthiases		
Ascariasis	B77	Yes
Hookworm	B76	Yes
Trichuriasis	B79	Yes
Onchocerciasis (river blindness)	B73	Yes
Cysticercosis/Taeniasis	B68–B69	Yes
Echinococcosis	B67	Yes
Filariasis	B74	Yes
Dracunculiasis (guinea-worm disease)	B72	No
Foodborne trematodiases		
Opisthorchiasis	B66.0	No
Clonorchiasis	B66.1	No
Fascioliasis	B66.3	Yes
Paragonomiasis	B66.4	No
**Bacteria**		
Leprosy	A30–B92	Yes
Trachoma	A71	Yes
Buruli ulcer^b^	A31.1	Unknown
Endemic treponema		
Yaws^c^	A66	Unknown
Pinta^d^	A67	Unknown
Endemic syphilis (Bejel)	A65	No
**Viruses**		
Rabies	A82	Yes
Dengue	A90–A91	Yes

ICD-10: *International statistical classification of diseases and related health problems*, 10th revision.

^a^ Presence of endemic areas (regions, states or municipalities).

^b^ Autochthonous cases reported, but endemicity is not determined.

^c^ Brazil was previously endemic for yaws, but the current status is unknown.

^d^ Cases reported in the past, but no cases have been reported since 1990.

### Analysis

For specific NTDs, we estimated average annual crude and age-adjusted mortality rates and 95% confidence intervals (CI). For all NTDs combined, we calculated crude, age-specific and age-adjusted mortality rates by sex, ethnicity and geographic area. Age-adjusted rates were calculated by the direct method based on the 2010 census. Age-specific rates were computed for the following age groups: 0–4, 5–9, 10–14, 15–19, 20–39, 40–59, 60–69 and older than 69 years. We included all data sets, even if information about some variables were not available in all cases. Details of missing data are presented in the tables.

We estimated (i) mortality rate ratios for all NTDs combined, by age, sex and ethnicity, based on the crude mortality rates; (ii) the proportion of all deaths attributed to NTDs; and (iii) the proportion of deaths from infectious and parasitic causes, (ICD-10 codes A00–B99), attributed to NTDs. For comparison, we also calculated deaths attributed to human immunodeficiency virus (HIV), tuberculosis and malaria.^20^

**Fig. 1:**
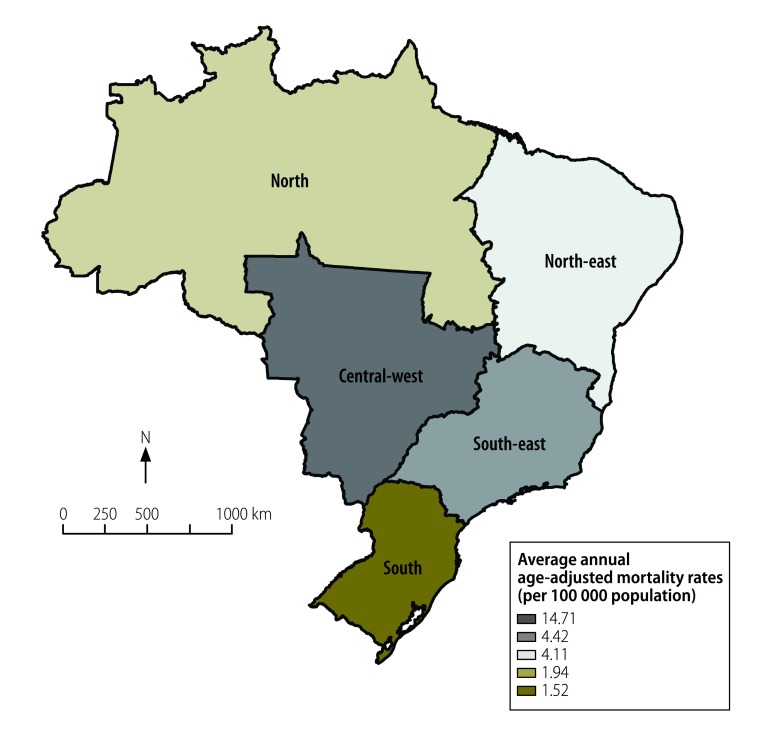
Annual average mortality rates from neglected tropical diseases in Brazil, 2000–2011

Note: Map produced using ArcGIS version 9.3 (ESRI, Redlands, CA, United States of America).

We used Stata version 11.2 (StataCorp LP, College Station, United States of America) for all analyses. The map of NTD mortality rates Fig. 1 was created using ArcGIS version 9.3 (ESRI, Redlands, United States of America). We used publicly available secondary data, which are anonymized to prevent identification of individuals. This study was approved by the Ethical Review Board of the Federal University of Ceará, Fortaleza, Brazil, registration number 751 109/2014.

Source of shapefile: Brazilian Institute of Geography and Statistics.

## Results

Between 2000 and 2011, 12 491 280 deaths were recorded. We identified 76 847 deaths with an NTD recorded as the underlying cause (Table 2). The average annual number of NTD-related deaths was 6404 (95% CI: 6238–6570), ranging from 6172 in 2001 to 6982 in 2008. Chagas disease was responsible for 58 928 deaths (76.7% of all deaths from NTDs), followed by schistosomiasis 6319 (8.2%) and leishmaniasis 3466 (4.5%). Deaths from NTDs were almost 60 times more frequent than from malaria (1288 deaths) and 1.3 times more frequent than from tuberculosis (59 281 deaths), but only 60% of the number of deaths from HIV (136 829) (Table 2).

**Table 2:** Mortality from neglected tropical diseases, by cause, Brazil, 2000–2011

Disease (ICD-10 code)	No. (% of total NTDs)	Median age (years)	Males (%)	Average annual no. of deaths	Notified deaths^a^ (%)	Deaths from infectious and parasitic diseases^b^ (%)	Crude mortality rates per 100 000 population per year (95% CI)^c^	Age-adjusted mortality rates per 100 000 population per year (95% CI)^d^
Chagas disease (B57)	58 928 (76.7)	65.6	57.3	4 910.7	0.47	10.55	2.65 (2.57–2.72)	3.37 (3.29–3.46)
Schistosomiasis (B65)	6 319 (8.2)	62.8	54.3	526.6	0.05	1.13	0.28 (0.26–0.31)	0.35 (0.33–0.38)
Leishmaniasis (B55)^e^	3 466 (4.5)	30.7	62.8	288.8	0.03	0.62	0.16 (0.14–0.17)	0.16 (0.14–0.18)
Dengue (A90–A91)	3 156 (4.1)	41.4	51.5	263.0	0.03	0.56	0.14 (0.13–0.16)	0.16 (0.14–0.17)
Leprosy (A30–B92)	2 936 (3.8)	64.2	72.1	244.7	0.02	0.53	0.13 (0.12–0.15)	0.16 (0.15–0.18)
Taeniasis/ cysticercosis (B68, B69)^f^	1 231 (1.6)	46.8	56.3	102.6	0.01	0.22	0.06 (0.05–0.07)	0.06 (0.05–0.08)
Soil-transmitted helminthiases (B76, B77, B79)^g^	518 (0.7)	2.7	46.5	43.2	NC	0.09	0.02 (0.02–0.03)	0.02 (0.01–0.03)
Rabies (A82)	113 (0.1)	14.7	65.5	9.4	NC	0.02	0.01 (0.00–0.01)	0.01 (0.00–0.01)
Echinococcosis (B67)	82 (0.1)	55.6	62.2	6.8	NC	0.01	NC	NC
Filariasis (B74)	66 (0.1)	59.6	40.9	5.5	NC	0.01	NC	NC
Human African trypanosomiasis (sleeping sickness) (B56)	9 (< 0.1)	64.7	36.4	0.8	NC	NC	NC	NC
Endemic treponematoses (A65, A66, A67)^h^	8 (< 0.1)	56.3	50.0	0.7	NC	NC	NC	NC
Onchocerciasis (river blindness) (B73)	5 (< 0.1)	2.2	60.0	0.4	NC	NC	NC	NC
Buruli ulcer (A31.1)	3 (< 0.1)	76.1	33.3	0.3	NC	NC	NC	NC
Dracunculiasis (guinea-worm disease; B72)	3 (< 0.1)	62.3	33.3	0.3	NC	NC	NC	NC
Trachoma (A71)	2 (< 0.1)	31.9	100.0	0.2	NC	NC	NC	NC
Foodborne trematodiases (B66.0, B66.1, B66.3, B66.4)^i^	2 (< 0.1)	56.1	50.0	0.2	NC	NC	NC	NC
**Total deaths from NTDs**	**76 847 (100.0)**	**63.8**	**57.6**	**6 403.9**	**0.62**	**13.75**	**3.45 (3.37–3.54)**	**4.30 (4.21–4.40)**
HIV (B20–B24)	136 829 (NA)	39.1	67.1	11 402.4	1.10	24.49	6.15 (6.04–6.26)	6.79 (6.67–6.91)
Tuberculosis (A15–A19)	59 281 (NA)	53.5	73.6	4 940.1	0.47	10.61	2.66 (2.59–2.74)	3.14 (3.06–3.22)
Malaria (B50–B54)	1 288 (NA)	32.9	62.6	107.3	0.01	0.23	0.06 (0.05–0.07)	0.06 (0.05–0.07)

CI: confidence interval; HIV: human immunodeficiency virus; ICD-10: *International statistical classification of diseases and related health problems*, 10th revision; NA: not applicable; NC: not calculated; NTDs: neglected tropical diseases.

^a^ Mortality by cause divided by the total number of deaths in the period (12 491 280 deaths).

^b^ Mortality by cause divided by deaths from infectious and parasitic diseases – ICD-10 codes A00-B99 (558 706 deaths).

^c^ Average crude mortality rates.

^d^ Mortality rates standardized to the 2010 Brazilian population.

^e^ Visceral leishmaniasis – B55.0: 2727; Cutaneous leishmaniasis – B55.1: 174; Mucocutaneous leishmaniasis – B55.2: 67; Leishmaniasis, unspecified – B55.9: 498.

^f^ Cysticercosis – B69: 2007 deaths; Taeniasis – B68: 13 deaths.

^g^ Ascariasis – B77: 827 deaths; Hookworm – B76: 25 deaths; Trichuriasis – B79: 1 death.

^h^ Yaws – A66: 23 deaths; Pinta – A67: 4 deaths; Bejel (endemic syphilis) – A65: 4 deaths.

^i^ Fascioliasis – B66.3: 4 deaths; Opisthorchiasis – B66.0: 1 death; Paragonimiasis – B66.4: 1 death; Clonorchiasis – B66.3: 0 deaths.

The median age at death from all NTDs combined was 63.8 years, (range: 0–108.5). Deaths from NTDs were most common in males (44 237/76 840; 57.6%); people older than 69 years (27 168/76 662; 35.4%); Caucasians (32 907/68 956; 47.7%); and residents in the south-east region (35 933/76 847; 46.8%). These deaths most commonly occurred in hospitals (55 791/76 629; 72.8%), followed by deaths at home (15 680/76 629; 20.5%). The median age of death was highest for chronic diseases such as Chagas disease, schistosomiasis and leprosy and lowest for soil-transmitted helminth infections, rabies, dengue fever and leishmaniasis (Table 2). The sex distribution also differed according to the disease; more than 70% (2117/2935) of leprosy deaths and 62.8% of leishmaniasis deaths (2177/3466) occurred in males (Table 2).

The average annual crude mortality rate was 3.45 deaths per 100 000 inhabitants (95% CI: 3.37–3.54), with an age-adjusted rate of 4.30 deaths per 100 000 inhabitants (95% CI: 4.21–4.40; Table 2 and Table 3). Average annual age-adjusted rates were significantly higher in males than females (Table 3). Age-specific rates increased with age, with 33.12 deaths per 100 000 inhabitants in people older than 69 years. Rates were 1.8 times higher in Afro-Brazilians compared to Caucasians (Table 3).

**Table 3:** Characteristics of people dying from neglected tropical diseases, Brazil, 2000–2011

Characteristic	No. (%) of deaths *n* = 76 847	Crude mortality per 100 000 population per year (95% CI)^a^	Age-adjusted mortality per 100 000 population per year (95% CI)^a,b^	RR^c^ (95% CI)
**Sex^d^**				
Female	32 603 (42.4)	2.89 (2.78–3.00)	3.63 (3.51–3.76)	1.00
Male	44 237 (57.6)	4.03 (3.90–4.16)	4.98 (4.84–5.13)	1.40 (1.33–1.47)
**Age group, years^d^**				
0–4	1 742 (2.3)	0.81 (0.69–0.95)	NC	1.00
5–9	472 (0.6)	0.22 (0.16–0.30)	NC	0.27 (0.19–0.38)
10–14	343 (0.4)	0.15 (0.10–0.22)	NC	0.19 (0.12–0.28)
15–19	446 (0.6)	0.19 (0.14–0.26)	NC	0.23 (0.16–0.34)
20–39	6 009 (7.8)	0.83 (0.76–0.90)	NC	1.02 (0.85–1.23)
40–59	22 835 (29.8)	5.50 (5.26–5.75)	NC	6.80 (5.75–8.06)
60–69	17 647 (23.0)	16.64 (15.81–17.51)	NC	20.59 (17.36–24.41)
≥ 70	27 168 (35.4)	33.12 (31.78–34.51)	NC	40.98 (34.65–48.47)
**Ethnicity^d^**				
Caucasian	32 907 (47.7)	3.01 (2.90–3.12)	NC	1.00
Afro-Brazilian	7 896 (11.5)	5.25 (4.86–5.67)	NC	1.75 (1.60–1.90)
Asian	334 (0.5)	1.96 (1.35–2.83)	NC	0.65 (0.45–0.94)
Mixed/Pardo Brazilian	27 695 (40.2)	3.13 (3.00–3.26)	NC	1.04 (0.98–1.10)
Amerindian	124 (0.2)	1.33 (0.73–2.43)	NC	0.44 (0.24–0.82)
**All**	76 847 (100.0)	3.45 (3.37–3.54)	4.30 (4.21–4.40)	NA

CI: confidence interval; NA: not applicable; NC: not calculated; RR: rate ratio.

^a^ Average annual crude- and age-adjusted mortality rates (per 100 000 inhabitants), calculated using the average number of deaths due to neglected tropical diseases as a numerator and population size in the middle of the studied period as a denominator. Population data on ethnicity was derived from the Brazilian National Censuses (2000 and 2010). Population size in relation to ethnicity for the middle of the period was derived from an average of the 2000 and 2010 censuses.

^b^ Age-standardized to the 2010 Brazilian population.

^c^ Based on crude mortality rates.

^d^ Data not available in all cases (sex: 7, age group: 185, and ethnicity: 7891).

Of the five regions, the central-west region had the highest age-adjusted rate (14.71 deaths per 100 000 inhabitants) and the southern region the lowest (1.52 deaths per 100 000 inhabitants; Fig. 1). The proportion of all deaths caused by NTDs was 0.62% (Table 2).

## Discussion

We have described mortality from NTDs in Brazil during a 12 year period. In general, NTDs with a predominantly chronic pathology showed the highest mortality. Chagas disease caused the highest number of deaths, followed by schistosomiasis and leishmaniasis, while leprosy also caused a considerable burden.

The high mortality from Chagas disease is a particular feature of Latin American countries, especially Brazil.^11^ During recent decades, there have been major efforts to reduce the burden of Chagas disease on the continent and transmission rates have been reduced considerably.^21^^,^^22^ However, because of the chronic nature of the disease, mortality rates will fall slowly.^11^^,^^23^

Brazil harbours most of the schistosomiasis burden in Latin America;^8^ the main endemic areas are in the north-east region of the country.^24^ Control programme measures implemented in recent decades were based mainly on periodical stool surveys in endemic areas, followed by treatment of positive cases. Consequently, morbidity and mortality from schistosomiasis have been reduced, but the disease has not been eliminated.^10^^,^^25^ Schistosomiasis control continues to be a challenge, with persistence and expansion of disease foci, even after years of integrated control measures.^25^^,^^26^ Internal migration of people, combined with the wide geographical distribution of intermediate snail hosts and poor sanitary conditions favour the permanence and establishment of new foci in Brazil.^25^

A considerable number of deaths were attributed to leishmaniasis, dengue fever and leprosy. Three forms of leishmaniasis – visceral, cutaneous, and mucocutaneous – differ in incidence, severity and geographic distribution in Brazil.^4^^,^^7^^,^^8^ Cutaneous leishmaniasis occurs in all 27 states, with most cases reported in the north region,^27^ whereas locally-transmitted cases of visceral leishmaniasis, the most serious form of the disease, are reported from 21 states, with the greatest burden in the north-east region.^9^^,^^28^^,^^29^ Visceral leishmaniasis is potentially fatal if not diagnosed and treated promptly^28^^,^^30^ and is responsible for most leishmaniasis deaths.^9^ There has been an increase in mortality from visceral leishmaniasis in Brazil in recent years. This is mainly due to the introduction of the disease into new geographic areas and host factors increasing case fatality rate, such as malnutrition, increasing age and immunosuppression, the latter being mainly due to HIV.^9^^,^^28^

Dengue fever has a wide geographic distribution and is also a national public health concern in Brazil.^31^ Despite intensified control measures in the country, in recent years there has been a steady increase in the number of dengue-related hospitalizations, severe cases and deaths.^15^^,^^32^ Increased geographical spread of the vector mosquitoes and the simultaneous presence of multiple dengue serotypes may partly explain the increases in severe dengue.^31^^,^^32^

The considerable number of leprosy deaths is surprising, since leprosy is usually seen as a disease with low case fatality.^14^^,^^33^^,^^34^ However, leprosy – even with continuously reduced new cases during the past decades – is an under-recognized cause of death.^33^ Based on the chronic nature of the disease and the transmission dynamics, deaths from leprosy will continue to occur for decades.

In general, age-adjusted NTD mortality rates were higher among males. This indicates gender-specific patterns of infectious disease exposure, as the relationship between gender and risk of infection is conditioned by different socioeconomic, environmental and behavioural factors.^10^^,^^11^^,^^32^ Males are less likely to seek early treatment, leading to increased morbidity and severity, which is particularly evident in the case of leprosy.^14^^,^^33^

For all NTDs combined, mortality rates increased with age and were highest among older age groups. This can be explained by the chronic nature of major NTDs with high mortality impact in Brazil, especially Chagas disease, schistosomiasis and leprosy.^10^^,^^11^^,^^33^^,^^35^ Interaction with chronic comorbidities which are common in these age groups, such as cardiovascular diseases, diabetes mellitus, hypertension and cancer, multiply the risk of severe disease and death.^36^ In people diagnosed with an NTD, possible co-infection with other NTDs and the presence of other chronic conditions should be assessed.^9^^,^^32^^,^^36^

Afro-Brazilians had higher NTD mortality rates compared with the Caucasian population. Similar to many other infectious diseases worldwide, this may be attributed to socioeconomic factors, poor housing, water and sanitation and reduced access to health care, which makes people vulnerable to neglected and poverty-related diseases in endemic areas.^11^^,^^33^ This pattern is also observed in other countries in Latin America and elsewhere.^37^^,^^38^

Our use of secondary mortality data leads to several limitations.^11^^,^^12^^,^^14^^,^^35^ Deaths may be underreported, despite recent progress in terms of the completeness and quality of mortality records.^9^^,^^10^ The proportion of deaths from ill-defined causes is distributed unequally between regions, urban and rural areas, age groups, and socioeconomic strata.^9^^,^^35^ In the year 2000, the proportion of deaths that were reported varied considerably, from 55.2% in Maranhão state in the north-east region to 100.0% in some states of the south and south-east regions. The coverage has improved steadily: in 2011, the regional differences were reduced, with the lowest coverage of 79.1%, also in Maranhão state.

Mortality from NTDs might be underestimated if underlying causes of death were coded as a pathology resulting from some NTDs, without mention of the infection that caused the pathology. For example, gastrointestinal bleeding, portal hypertension and oesophageal varices may be caused by schistosomiasis and Chagas disease can cause heart failure.^10^^,^^36^^,^^39^ We could have included certificates where NTDs were recorded as cause of death in any part of the death certificate rather than only as the underlying cause. However, we opted to present an analysis based on the underlying causes of deaths as this is the usual standard applied in mortality data analysis.^23^^,^^35^ Analysis by ethnicity is limited by missing data.

We conclude that NTDs continue to be an important public health problem in Brazil. There is a need to improve integrated control measures in the areas with the highest morbidity and mortality burden. Specific disease control programmes for diseases which are usually considered of chronic nature and not a cause of death, should also take case-fatality rates into account.
